# A Whole-Spine Radiography Study to Reduce Patient Exposure Dose and Artifacts Using the EOS Imaging System

**DOI:** 10.3390/bioengineering11090863

**Published:** 2024-08-23

**Authors:** DongHee Hong, YoungCheol Joo, Eunhye Kim

**Affiliations:** 1 Department of Radiological Science, Shinhan University, Uijeongbu 11644, Gyeonggi-do, Republic of Korea; hansound2@shinhan.ac.kr; 2 Department of Radiology, Samsung Medical Center, Seoul 06351, Republic of Korea; jumyself.joo@samsung.com; 3 Department of Radiological Science, Hanseo University, Seosan 31962, Republic of Korea

**Keywords:** whole-spine radiography, entrance surface dose, morphotype, scan speed, X-ray tube cooling time, EOS

## Abstract

Whole-spine radiography can be accomplished through two methods: (1) segmented imaging employing X-ray tube angulation and detectors, or (2) the Euronext Paris Advanced Orthopedic Solutions (EOS) 2D Imaging system that can capture the entire spine in a single image using X-ray tubes and detectors oriented at a 90-degree angle. This study aimed to establish optimal EOS examination parameters based on patient morphotype and scan speed to reduce patient radiation exposure, repeat examinations, heat stress on equipment, and X-ray tube cooling time. X-ray exposure conditions involved adjustments of scan speed ranging from two to four steps, contingent upon the patient’s morphotype (‘S’, small body; ‘M’, medium body; and ‘L’, large body. Patient dose measurements were conducted 20 times for each set of conditions. When transitioning from an ‘S’ to an ‘M’ morphotype at a constant scan speed, the entrance skin dose (ESD) exhibited an increase of approximately 41.25 ± 4.57%. A similar change from an ‘M’ to an ‘L’ morphotype resulted in an ESD increase of roughly 59.56 ± 24.00%. A transition from an ‘S’ to an ‘L’ morphotype at the same scan speed manifested an ESD elevation of approximately 124.21 ± 26.96%. This study underscores significant variations in radiation dose, ranging from 40% to 50%, when altering morphotype while maintaining a consistent scan speed.

## 1. Introduction

The spine and pelvis play a pivotal role in maintaining human body balance, with spinal sagittal alignment and pelvic parameters being crucial for diagnosing spinal and hip-joint disorders [1,2,3]. Whole-spine radiography conducted in a standing position to reflect real-life conditions with gravitational forces is instrumental in observing these factors [4,5,6,7,8]. Currently, standing whole-spine radiography (WSR) can be executed using two distinct methodologies. One approach employs the X-ray tube angulation method along with detectors to capture segmented portions of the spine, subsequently piecing them together. The second method utilizes a detector capable of acquiring a single image of the entire spine, with X-ray tubes and detectors oriented at a 90-degree angle, where the X-ray tube and detector move vertically to simultaneously capture images. This method is known as the Euronext Paris Advanced Orthopedic Solutions (EOS) 2D imaging system (EOS Imaging, Paris, France) [9,10,11].

The EOS imaging system allows for concurrent acquisition of posteroanterior (PA) and lateral projections of the entire spine in the standing position, obviating the need for the patient to change positions [1,9,12,13]. It employs a fan-shaped radiation beam that can scan the body vertically along the sagittal axis, thereby minimizing image distortion compared to conventional techniques [14,15,16,17]. Due to the advanced technology of the EOS equipment, it can produce images of comparable quality while utilizing lower radiation doses in contrast to the conventional whole-body radiography system, where radiation doses are 5–9 times higher [4,18,19,20,21]. Consequently, the EOS imaging system has emerged as a valuable option for diagnosing spinal and hip-joint disorders and prioritizing patient safety.

The EOS imaging system customarily adjusts examination conditions based on the patient’s body type, termed “morphotype”, and scan speed, often with application of an appropriate physical filter (copper or aluminum) [22,23]. The morphotype classification includes ‘S’ (small body), ‘M’ (medium body), and ‘L’ (large body), while scan speed ranges from 1 to 10, with higher values signifying more rigorous examination conditions. The manufacturer typically recommends employing the ‘M’ body type in conjunction with a scan speed of 3 or 4.

However, most patients undergoing EOS are patients with pain or elderly patients with spinal and hip diseases. It is very difficult for them to maintain a good posture with straight waist and legs. Therefore, shortening the examination time is very important in terms of preventing repeat exposure, reducing patient exposure doses, reducing the heat stress on equipment, and improving efficiency of operation. Since EOS irradiates X-rays continuously (8–20 s or more) from the start to the end of the examination, an X-ray tube with a heating rate higher than the one used in general X-ray development devices is used.

This study determined if a combination of body size and examination speed could reduce patient’s exposure dose and motion or artifacts by shortening the examination time during spinal full-length radiation using EOS. This study aimed to minimize patient’s exposure dose and X-ray tube cooling time. A method suitable for patient’s examination environment by combining EOS examination conditions is suggested.

## 2. Materials and Methods

**Figure 1:**
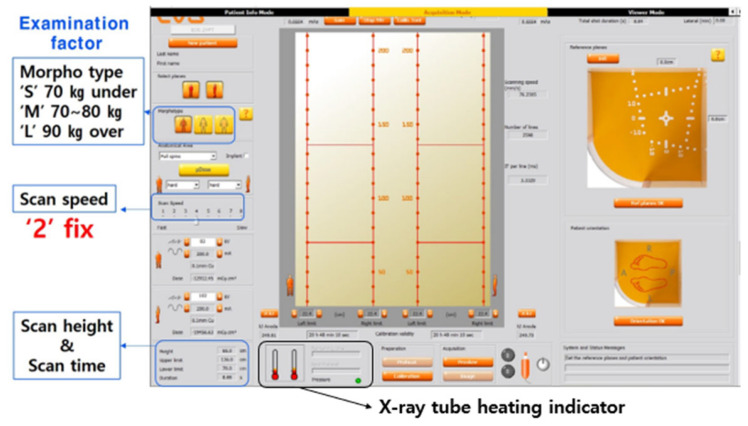
Examination factors of EOS imaging system, including morphotype, scan speed, scan height and time, and X-ray tube heating indicator.

This study utilized the Alderson Rando Woman Phantom (Radiology Support Devices, Long Beach, CA, USA) as a human tissue-equivalent material. The X-ray generator employed was an EOS imaging system (Biospace Imaging, Paris, France). Entrance Skin Dose (ESD) is a measurement of the radiation dose absorbed by the skin when radiation reaches a patient. It is typically measured using a dosimeter. In this study, ESD was measured using an Unfors PSD (Unfors Instruments Inc., New Milford, CT, USA). In general, EOS determines examination conditions using morphotype and scan type. Accordingly, kV, mA, and filter of examination conditions under the scan speed are changed to values set by the equipment company (Figure 1). Of course, the user can adjust the kV and mA. However, this is a user’s subjective manipulation. It is generally not used in clinical practice. It is not recommended by equipment companies.

**Figure 2:**
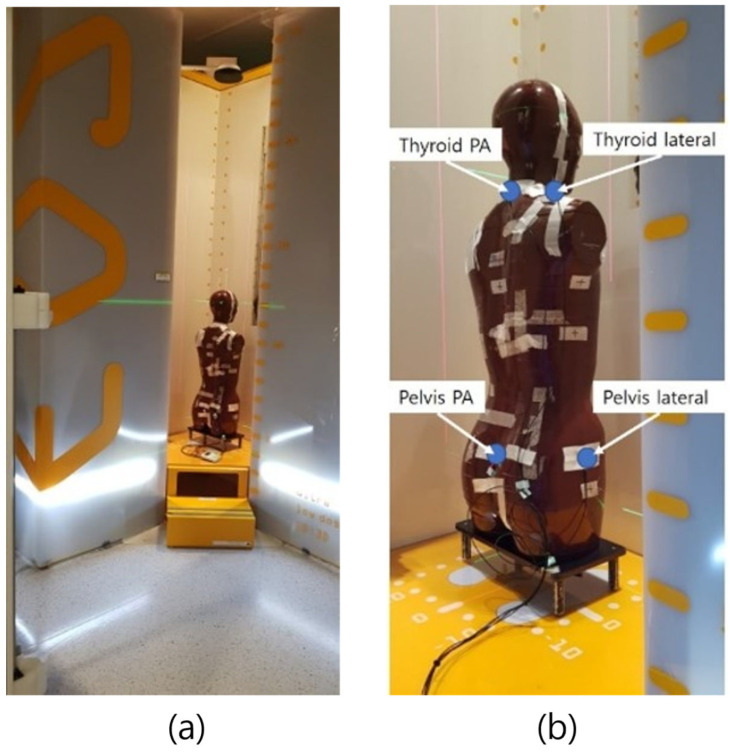
Phantom posture and dosimetry position: (**a**) The phantom was placed in the PA posture and the scan range was from the frontal sinus to the proximal femur. (**b**) Dosimeters were attached to the back and sides at the level of thyroid and pelvic cavities.

**Figure 3:**
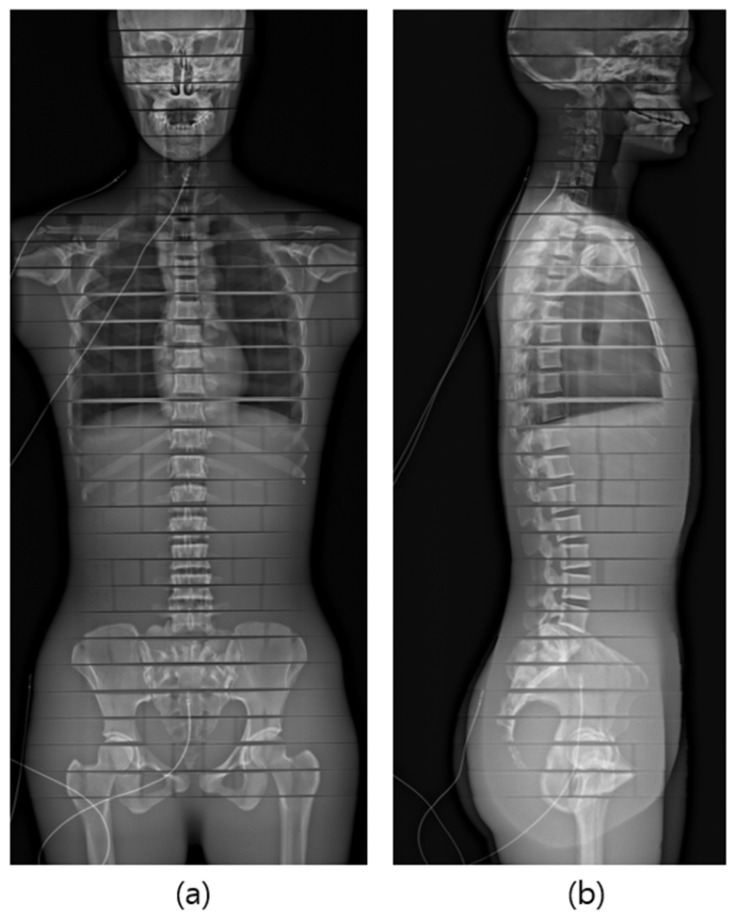
Radiograph of phantom from EOS examination. (**a**) PA posture, (**b**) Lateral posture.

The phantom was meticulously positioned at the center of the equipment in the posteroanterior (PA) orientation, aligning the anterior–posterior surface of the phantom with the equipment’s guidance lines. The X-ray scanning range encompassed the entire region, extending from the height that included frontal sinuses to the proximal femur (Figure 2 and Figure 3).

### 2.1. Investigation Conditions and Dosage Measurement Method

X-ray irradiation conditions which are kV and mA values refer to values set in the EOS equipment. The exposure time varied depending on the morphotype and scan speed as shown in Table 1, resulting in a change in the overall evaluation of the dose. These were adjusted across three exposure speed levels categorized as ‘S’ (small body, under 70 kg), ‘M’ (medium body, between 70 to 80 kg), and ‘L’ (large body, over 90 kg). Twenty images were acquired under each condition, with specific scanning conditions detailed in Table 1. The exposure speed can be set from level 1 to level 10. The fastest scan speed is 1 and the slowest setting is 10, which means that the tube and the detector move quickly. The amount of information they capture decreases, while the amount of heat the tube receives decreases. Therefore, the equipment company recommends using a scan speed of 3 or 4. However, in this study, we would like to find out conditions when using a scan speed of 2, considering that it is difficult for a patient to maintain the position for the radiograph.

The total scan time per image capture varies depending on the patient’s height if the scan speed is set equally. On average, about 8 to 9 s are required for a scan speed of 2, 12 to 13 s for a scan speed of 3, and 16 to 17 s for a scan speed of 4. This is the time when X-rays are irradiated. Compared to when general radiation equipment acquires an image in one irradiation, EOS requires a very high heat capacity because X-rays are continuously irradiated for the above time, which is proportional to the scan time. If the scan time increased, it would be difficult to maintain the patient’s posture while obtaining a radiograph which could result in the patient’s motion and image artifacts. It would also increase heat stress due to an increase in scan time and increase load on the tube. Patient irradiation dose was gauged by employing semiconductor dosimeters affixed to the back and sides of the phantom at thyroid and pelvic cavity levels. Both anteroposterior (PA) and lateral (Lat.) radiograph images were acquired. ESD for each condition was recorded.

### 2.2. Data Analysis Method

For data analysis, ESD measurements according to changes in scan time for each morphotype were tested for data normality through the Shapiro–Wilk test (*p* > 0.05). Dose values measured according to changes in scan time for each morphotype are described as mean, standard deviation, minimum, and maximum values. One-way analysis of variance (ANOVA) was performed for comparative analysis of average values between scan time groups for each morphotype. Duncan’s test was used for post hoc analysis. SPSS version 22.0 (SPSS Inc., Chicago, IL, USA) was used for all statistical analyses. The significance level (α) was set to be 0.05 and significance probability (*p*) was set to be 0.05 or less.

## 3. Results

In the context of body size ‘S’, ESD values for the thyroid position ranged from 105.81 ± 1.10 to 216.35 ± 3.17 μGy for PA radiograph images and from 146.17 ± 0.78 to 293.65 ± 0.84 μGy for lateral radiograph images. Notably, both sets of measurements demonstrated statistically significant variations in average values (*p* < 0.01). In the case of the pelvic region, ESD values spanned from 81.79 ± 0.71 to 162.31 ± 1.54 μGy for PA radiograph images and from 133.65 ± 0.82 to 266.40 ± 1.20 μGy for lateral radiograph images. ESD values also exhibited substantial variations across different scanning speeds (*p* < 0.01). Post hoc analysis discerned distinct groupings for both anatomical regions (Table 2).

For body size ‘M’, at a scanning speed of 2 to 4, average dose values for the thyroid position ranged from 152.72 ± 1.15 to 308.74 ± 1.16 μGy for PA radiograph images and ranged from 203.32 ± 0.92 to 405.51 ± 1.13 μGy for lateral radiograph images. Significantly divergent average values were observed for both PA and lateral radiograph images (*p* < 0.01), with post hoc analysis revealing distinct groupings for all conditions. In the pelvic region, average ESD values ranged from 119.20 ± 0.85 to 238.59 ± 2.33 μGy for PA radiograph images and from 180.82 ± 0.69 to 361.94 ± 1.12 μGy for lateral radiograph images. Average values displayed statistically significant differences among various groups (*p* < 0.01). Post hoc analysis elucidated discrete groupings for each condition (Table 2).

In the case of body size ‘L’, the average ESD ranged from 224.20 ± 0.85 to 450.28 ± 1.44 μGy for thyroid PA radiograph images and ranged from 350.26 ± 1.48 to 699.90 ± 0.90 μGy for thyroid lateral radiograph images. Statistically significant differences in average values were observed between groups. Post hoc analysis discerned distinct classifications (*p* < 0.01). In the pelvic region, the ESD ranged from 154.81 ± 0.91 to 309.53 ± 1.62 μGy for PA radiograph images and ranged from 341.63 ± 0.78 to 688.72 ± 0.97 μGy for lateral radiograph images. Measured values demonstrated statistically significant variations (*p* < 0.01). Post hoc analysis also identified separate groupings for each condition (Table 2).

In the EOS device, unlike the X-ray exposure of a general X-ray generator, X-rays are exposed throughout the exposure range. As a result, the amount of heat received by the X-ray tube is higher than that of a general X-ray generator. Investigation conditions recommended by the equipment manufacturer require a certain period of time to cool the X-ray tube after performing procedures for three to four patients. This requirement for continuous cooling time due to scan speed use errors creates difficulties in the smooth operation of an examination room with the EOS equipment.

## 4. Discussion

Whole-body radiography (WBR) stands as the sole radiographic method that affords a view of the sagittal balance of the axial skeletal system while considering gravitational effects on the human body. Sagittal balance of the spine is vital for proper gravity distribution and reduced energy consumption. Alterations of this balance due to degenerative changes, structural abnormalities, incorrect habits, and other factors related to the spine and pelvis can lead to back pain [5,6,7,8]. It has been estimated that 70–90% of individuals experience back pain at least once in their lifetime [24,25,26,27].

The EOS imaging system is renowned for its capability to provide imaging with minimal distortion of anatomical structures and the lowest radiation exposure [28,29,30,31]. Although there is no significant difference in image quality between a conventional digital radiography (DR) system and the EOS imaging system, the radiation dose to the thyroid gland with EOS is 15.5 times lower than that of the DR system, even lower with a micro-dose EOS, ranging from 5.9 to 27.0 times lower [32]. A previous study comparing ESD using EOS and DR systems for pediatric patients has indicated that EOS has a slightly higher ESD for the cervical region (172.8 μGy) than the DR system (122.9 μGy), while the sacral region has a 2.7-fold higher dose with the DR system (EOS: 189 μGy, DR system: 524.6 μGy) [33]. The EOS allows for a shorter examination time (EOS: 248 s, DR system: 449 s) for full spine examination, with lower ESD (EOS: 158.4 ± 103.8 cGy × cm^2^) than the DR system [34]. These prior studies underscore the advantage of EOS for whole-body radiography with lower radiation exposure than conventional methods [30,31,33,34,35,36].

However, EOS differs from conventional X-ray devices because patients are exposed to X-rays over the entire examination range [32,33]. Since the X-ray tube is used for patients for a longer period of time compared to a conventional X-ray generator, it not only increases the overall exposure dose, but also increases the time for patients to take pictures without moving [32,33]. This time delay brings pain to elderly patients and patients with back pain. It creates motion or artifacts by movement. In addition, repeated examinations can put a high heat load on the X-ray tube and lead to difficulties in efficient work in the radiology department

When transitioning from ‘S’ to ‘M’ at the same examination speed, the ESD increase was 41.25 ± 4.57%. Similarly, the shift from ‘M’ to ‘L’ and from ‘S’ to ‘L’ resulted in ESD increases of 59.56 ± 24.00% and 124.21 ± 26.96%, respectively. Furthermore, within the same body type, altering the examination speed revealed a consistent pattern, yielding approximately 50% increase in ESD when transitioning from examination speed 2 to 4. X-ray exposure time is approximately 8–10 s for speed 2, 15–17 s for speed 3, and 18–20 s for speed 4. Given that most patients undergoing EOS examination are patients with pain or elderly and suffer from degenerative spinal conditions, it is challenging for them to maintain a static posture without movement for 8–20 s. Hence, acquiring images swiftly during EOS examination is essential. This study explored the interplay between body type and examination speed to facilitate examinations with comparable patient exposure doses while minimizing delays.

The findings of this study revealed that when comparing ESD in thyroid and pelvic regions for PA and lateral radiograph views, a similar dose group emerged between body type ‘S’ and examination speed 3 as well as between body type ‘M’ and examination speed 2. A similar dose group was also observed between body type ‘S’ and examination speed 4, between body type ‘M’ and examination speed 3, and between body type ‘L’ and examination speed 2. However, distinctions in exposure dose based on location were noted between body type ‘M’ and examination speed 4 as well as between body type ‘L’ and examination speed 3. These findings underscore that, rather than increasing examination speed for the same body type, reducing ESD can be achieved by altering body type and decreasing the examination speed. This approach proves effective in minimizing examination time and X-ray tube heat, consequently reducing X-ray tube cooling time.

This study has some limitations. First, it was conducted with only one type of phantom and dosimeter. In addition, it did not conduct a quantitative evaluation of image quality for each condition. However, orthopedic spine specialists who evaluated the diagnostic value of the image concluded that the combination of test conditions claimed in this study did not interfere with qualitative evaluation of the image, considering the purpose of WBR testing.

## 5. Conclusions

The EOS system automatically sets tube voltage, tube current, and additional filters based on the combination of morphotype and scan speed. This study investigated changes in patient dose according to different combinations of morphotype and scan speed, aiming to propose optimal combinations of body type and examination speed for EOS examinations. The goal was to minimize patient exposure dose and artifacts from motion. The findings of this study indicated that altering the body type at a constant examination speed resulted in exposure dose variations of 40–50% and that increasing the examination speed by 1 at the same body type led to an approximate 50% elevation in exposure dose. Therefore, reducing the examination speed for patients with the same body type whenever possible is needed to reduce patient dose, alleviate discomfort experienced by patients from maintaining a position, and improve examination efficiency by shortening the examination time. For full-spine EOS examinations, it is recommended to elevate the body type by one level while maintaining the examination speed at 2 as a more efficient approach for reducing examination duration as opposed to increasing the examination speed within the same body type. It would not only help patients by decreasing examination duration, but also help radiologic technologists to remove motion artifacts from patients to improve image quality.

## Figures and Tables

**Table 1:** The exposure condition of each morphotype and scan speed used in this study.

Morphotype	Scan Speed	Tube Side	kV	mA	Exposure Time (sec)	Filter
‘S’	2	frontal	83	200	8.06	0.1 mm Cu
		lateral	102	200		
	3	frontal	83	200	12.10	
		lateral	102	200		
	4	frontal	83	200	16.13	
		lateral	102	200		
‘M’	2	frontal	90	250	8.06	
		lateral	105	250		
	3	frontal	90	250	12.10	
		lateral	105	250		
	4	frontal	90	250	16.13	
		lateral	105	250		
‘L’	2	frontal	100	250	8.06	0.1 mm Cu
		lateral	110	320		1 mm Al
	3	frontal	100	250	12.10	0.1 mm Cu
		lateral	110	320		1 mm Al
	4	frontal	100	250	16.13	0.1 mm Cu
		lateral	110	320		1 mm Al

**Table 2:** Results of comparing average ESD values for thyroid PA location according to each scanning speed for morphotype ‘S’, ‘M’, ‘L’.

Body Part Location		Scan Speed	n	Mean ± SD (μGy)	Min	Max	F	*p*
Thyroid ‘S’	PA	2	20	105.81 ± 1.10	104.3	108.5	13,168.312	0.01
		3		157.83 ± 1.63	155.9	161.5		
		4		216.35 ± 3.17	211.4	224.3		
	Lateral	2		146.17 ± 0.78	145.1	147.9	21,284.089	0.01
		3		220.02 ± 0.78	218.6	221.7		
		4		293.65 ± 0.84	292	294.8		
Pelvis ‘S’	PA	2		81.79 ± 0.71	80.68	82.99	168,243.685	0.01
		3		122.27 ± 1.28	120.4	124.8		
		4		162.31 ± 1.54	160.2	165.1		
	Lateral	2		133.65 ± 0.82	132	134.8	80,576.104	0.01
		3		200.22 ± 1.07	197.6	201.7		
		4		266.40 ± 1.20	264.3	269.3		
Thyroid ‘M’	PA	2	20	152.72 ± 1.15	150.6	155.4	115,882.607	0.01
		3		230.39 ± 0.69	229.4	231.6		
		4		308.74 ± 1.16	306.1	310.8		
	Lateral	2		203.32 ± 0.92	201.5	204.6	27,910.241	0.01
		3		305.16 ± 0.92	303.2	307		
		4		405.51 ± 1.13	403.4	407.6		
Pelvis ‘M’	PA	2		119.20 ± 0.85	117.7	120.6	204,927.239	0.01
		3		177.19 ± 1.22	174.5	179.3		
		4		238.59 ± 2.33	234.6	242.5		
	Lateral	2		180.82 ± 0.69	179.3	182.1	168,865.094	0.01
		3		271.66 ± 1.07	269.7	272.1		
		4		361.94 ± 1.12	360.1	364.7		
Thyroid ‘L’	PA	2	20	224.20 ± 0.85	222.5	225.8	205,655.158	0.01
		3		336.66 ± 0.95	334.5	338.1		
		4		450.28 ± 1.44	447.9	453.4		
	Lateral	2		350.26 ± 1.48	347	352.4	77,874.666	0.01
		3		525.10 ± 1.31	523.9	528.7		
		4		699.90 ± 0.90	698.3	701.4		
Pelvis ‘L’	PA	2		154.81 ± 0.91	153.5	156.5	387,869.836	0.01
		3		231.37 ± 1.06	229.4	233.5		
		4		309.53 ± 1.62	306.7	313.1		
	Lateral	2		341.63 ± 0.78	339.4	342.6	635,028.568	0.01
		3		515.10 ± 1.12	513.1	517.7		
		4		688.72 ± 0.97	687.1	690.3		

## Data Availability

Data are contained within the article.
